# HolomiRA: a reproducible pipeline for miRNA binding site prediction in microbial genomes

**DOI:** 10.1186/s12859-025-06241-x

**Published:** 2025-10-01

**Authors:** Jennifer Jessica Bruscadin, Tainã Figueiredo Cardoso, Liliane Costa Conteville, Juliana Virginio da Silva, Adriana Mércia Guaratini Ibelli, Gabriel Alexander Colmenarez Pena, Thanny Porto, Priscila Silva Neubern de Oliveira, Bruno Gabriel Nascimento Andrade, Adhemar Zerlotini, Luciana Correia de Almeida Regitano

**Affiliations:** 1 Center of Biological and Health Sciences, Federal University of São Carlos, São Carlos, São Paulo Brazil; 2 Embrapa Southeastern Livestock, São Carlos, São Paulo Brazil; 3 Munster Technological University, Cork, Ireland; 4 Embrapa Digital Agriculture, Campinas, São Paulo Brazil

**Keywords:** Holobiome, Host–pathogen interaction, Gene regulation

## Abstract

**Background:**

Small RNAs, such as microRNAs (miRNAs), are candidates for mediating communication between the host and its microbiota, regulating bacterial gene expression and influencing microbiome functions and dynamics. Here, we introduce HolomiRA (Holobiome miRNA Affinity Predictor), a computational pipeline developed to predict target sites for host miRNAs in microbiome genomes. HolomiRA operates within a Snakemake workflow, processes microbial genomic sequences in FASTA format using freely available bioinformatics software and incorporates built-in data processing methods. The pipeline begins by annotating protein-coding sequences from microbial genomes using Prokka. It then identifies candidate regions, evaluates them for potential host miRNA binding sites and the accessibility of these target sites using RNAHybrid and RNAup software. The predicted results that meet the quality filter parameters are further summarized and used to perform a functional analysis of the affected genes using SUPER-FOCUS software.

**Results:**

In this paper, we demonstrate the use of the HolomiRA pipeline by applying it to publicly available metagenome-assembled genomes obtained from human feces, as well as from bovine feces and ruminal content. This approach enables the prediction of bacterial genes and biological pathways within microbiomes that could be influenced by host miRNAs. It also allows for the identification of shared or unique miRNAs, target genes, and taxonomies across phenotypes, environments, or host species.

**Conclusions:**

HolomiRA is a practical and user-friendly pipeline designed as a hypothesis-generating tool to support the prediction of host miRNA binding sites in prokaryotic genomes, providing insights into host-microbiota communication mediated by miRNA regulation. HolomiRA is publicly available on GitHub: https://github.com/JBruscadin/HolomiRA.

**Supplementary Information:**

The online version contains supplementary material available at 10.1186/s12859-025-06241-x.

## Background

MicroRNAs (miRNAs) are non-coding RNAs that post-transcriptionally regulate gene expression in eukaryotes [1]. Recent studies suggest that miRNAs may contribute to host-microbiota communication, although the mechanisms remain unclear. Evidence indicates that miRNAs can be secreted via extracellular vesicles (EVs) [2] and, in some cases, may enter microbial cells, modulate microbial gene expression, and shape host-microbiome interactions [3–5]. Some studies have described that bacteria can modulate host miRNA expression for their own benefit [6, 7]. However, evidence for a bidirectional relationship between the microbiome and host miRNAs continued to grow.

In 2016, Liu et al. [4] demonstrated that human miRNAs, such as hsa-miR-1226-5p and hsa-miR-515-5p, can be internalized by *Escherichia coli* and *Fusobacterium nucleatum* after co-incubation, while variability in bacterial uptake of miRNAs was also observed. Furthermore, miR-30d regulated the expression of a lactase in *Akkermansia muciniphila*, leading to increased *Akkermansia* abundance in the gut. The expanded *Akkermansia* population, in turn, promoted regulatory T cell development, and suppressed autoimmune encephalomyelitis symptoms in mice [8]. Recently, Wang et al. [9] showed that hsa-miR-7704 inhibited the growth and adhesion of *Bifidobacterium longum* by suppressing the *proB* gene. Eukaryotic miRNAs can influence bacterial gene expression at the post-transcriptional level, thereby affecting the composition of the microbial community [10], and/or can enter bacterial cells through endocytosis, specifically regulating bacterial gene transcripts and influencing bacterial growth [11].

In prokaryotes, the translation of specific mRNAs is partially controlled by small RNAs (sRNAs), which typically bind to the 5’ UTR of mRNA. This region includes the Shine-Dalgarno (SD) sequence, a ribosome binding site (RBS) in prokaryotic mRNA upstream of the start codon [12]. These sequences can extend from twenty nucleotides upstream to fifteen nucleotides downstream of the translation initiation codon [13]. In the most frequent scenario, sRNAs prevent the binding of the 30S ribosomal subunit to the RBS region, promoting translation repression and degradation of the target mRNA [14]. Although such a mechanism of miRNA regulation in prokaryotic genomes is not fully described, it is hypothesized to be similar to that of sRNAs. One hypothesis is that eukaryotic miRNAs may bind to mRNAs harboring weak SD sequences, where the ribosome assembly rate is slow, thus enhancing the chance for miRNA-mRNA interaction [10].

Current research on prokaryotic gene targets of host-derived miRNAs primarily relies on sequence-based analysis and RNA thermodynamics. However, to date, no dedicated *in silico* tool specifically tailored for this prediction has been developed. Consequently, existing tools and software packages designed for intraspecies alignment (such as BLASTn and miRBase) and hybridization analysis (such as RNAhybrid and miRanda) have been adapted to investigate interspecies interactions [4, 8, 15, 16], and focus on directly identifying of the target genome with miRNAs of interest. In addition, research in eukaryotic systems has highlighted the critical role of structural accessibility in enabling miRNA binding. Consequently, target prediction approaches may benefit from incorporating this factor, which can be evaluated by calculating the total binding free energy [10].

Therefore, aiming to expand our understanding of the functional role of miRNAs in host-microbiome communication, we developed a user-friendly computational pipeline to identify putative interactions between host miRNAs and genomes from their microbiota, which can be applied to any prokaryotic genome. The Holobiome miRNA Affinity Predictor (HolomiRA) uses publicly available tools for sequence annotation, miRNA target prediction, target accessibility, and enrichment analysis of impacted genes. To our knowledge, this is the first pipeline designed to test interactions between host miRNAs and microbiome genomes, representing a significant contribution toward understanding host regulatory influences on microbiome activity.

## Implementation

### HolomiRA pipeline

**Fig. 1:**
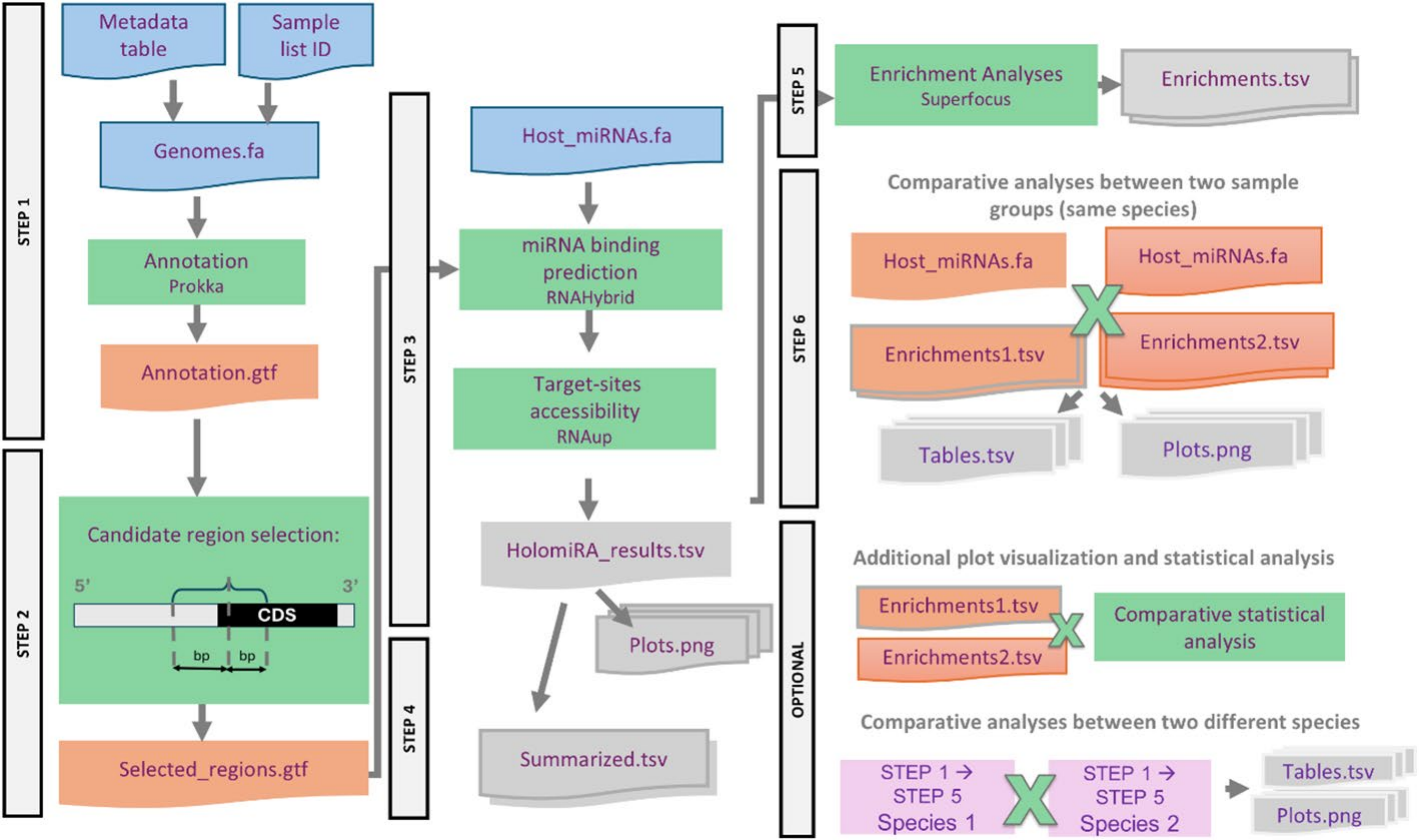
Schematic representation of the HolomiRA pipeline. CDS = Coding DNA Sequence; MFE = Minimum Free Energy. The pipeline requires four initial files (highlighted in blue): 1) a “Genomes.fa” file containing any prokaryotic genome, 2) a “Host_miRNAs.fa” file containing any collection of mature host miRNA sequences, 3) a sample identification list, and 4) a metadata file describing each microbial genome (*e.g.,* taxonomic classification, environment of origin, etc.). All other steps are automated. The comparative analysis between sample groups (Step 6) is executed only if more than one sample category (*e.g.,* phenotype, case–control, environment) is specified in the config.yaml file

HolomiRA is an accessible, user-friendly, and reproducible pipeline (Fig. 1) for identifying host miRNA binding sites within microbial genomes, based on the growing evidence of microbiota regulation by miRNAs. HolomiRA requires Snakemake (version ≥ 7.32.3) and Conda (version ≥ 24.1.0) to manage software dependencies. The workflow automates the installation and configuration of the correct software versions. This approach mitigates potential version conflicts and enhances usability, facilitating adoption by researchers with varying levels of experience in bioinformatics and data analysis.

HolomiRA requires four mandatory input files: i) microbial genome sequences in FASTA format, ii) a FASTA file containing mature host miRNA sequences, iii) a sample identification list, and iv) a metadata file containing additional information about each microbial genome, including its taxonomic classification and environmental source (*e.g*., tissue type, species, phenotype, etc.). Analysis parameters are defined within a YAML configuration file, where users can adjust biological thresholds such as: i) the number of upstream and downstream base pairs flanking the CDS start site to define the candidate target region, ii) parameters for miRNA hybridization filtering (*i.e.,* seed position, minimum free energy [MFE], and p-value thresholds), and iii) the total ΔG value, which represents the final interaction energy used to assess miRNA-target accessibility. The following subsections describe each analytical step implemented in HolomiRA.

Step 1 – Predict coding sequences from microbial genomes.

HolomiRA requires genomic DNA sequences in FASTA format as input. These sequences are annotated using Prokka (v. 1.14.6) [17], which first identifies the coordinates of protein-coding genes using Prodigal (v. 2.6.3) [18]. Subsequently, by default, Prokka compares the predicted sequences against various public databases, such as RefSeq, UniProt, and Pfam [17], to provide accurate and reliable functional annotations for each predicted protein.

Step 2 – Select candidate target regions.

The annotated coding sequences (CDS) are extracted from the annotation files generated by Prokka. The 5'-most nucleotide coordinate of each CDS region is used to define a genomic window that delimits candidate regions for miRNA-binding site screening. By default, these windows span from −15 to + 20 nucleotides relative to the CDS start, based on the typical extent of RBS regions [13]. Candidate sequences within these windows are then extracted from the genomes and used as input for miRNA target site prediction. As a conservative filtering step, HolomiRA includes only CDS longer than 150 nucleotides, aiming to minimize potential artifacts associated with very short sequences.

Step 3 – Search for host miRNAs binding sites in the candidate regions and evaluate their accessibility.

These steps rely on RNAhybrid (v. 2.1.2) [19] and RNAup from the ViennaRNA package (v. 2.5.1) [20]. The combined use of RNAhybrid and RNAup offers a robust approach for predicting miRNA-mRNA interactions by integrating the assessment of binding affinity with the structural accessibility of the target region. RNAhybrid efficiently screens for potential interactions based on hybridization energy, identifying candidates with high thermodynamic stability. RNAup then refines these predictions by considering the mRNA's secondary structure, calculating the energy required to expose the target region and allow duplex formation with the miRNA. This sequential strategy aims to enhance the biological plausibility of predictions by considering both hybridization energy and the estimated accessibility of target sites.

First, RNAhybrid [19] identifies the most energetically favorable hybridization sites between each miRNA sequence and the selected CDS window sequence. This step uses as input the CDS window sequence obtained in Step 2, and the mature host miRNA sequences, which can be obtained from public databases such as miRDB [21], miRBase [22], and MirGeneDB [23]. Unlike eukaryotic miRNA-target interactions, bacterial sRNAs typically exhibit longer seed regions than miRNAs [24–26]. This suggests that restricting the sequence comparison to the seed sequence may overlook potential bacterial gene targets. By default, HolomiRA uses the entire mature miRNA sequence to search for target genes. In this step, HolomiRA considers a p-value threshold < 0.01 and a MFE <  −20 kcal/mol as cut-offs for filtering results.

After this initial screening, a window of + 150 nt is constructed around the original CDS window, and the interaction between this extended region and the candidate miRNA is evaluated using RNAup [20] to analyze target site accessibility. By default, HolomiRA performs the final filtering of candidate sequences by applying a total ΔG (Gibbs free energy of binding between two RNAs) threshold of <  −15 kcal/mol.

Step 4 – Summarizing and visualizing prediction results.

To facilitate the interpretation of the predicted host miRNA binding sites, HolomiRA provides multiple output formats. These include: i) a comprehensive results table, ii) a summary table grouped by miRNA, and iii) a summary table grouped by microbial genome. Additionally, HolomiRA generates bar plots summarizing the data and ranking miRNAs according to the number of predicted interactions with genomes and genes.

Step 5 – Functional enrichment analysis of putative impacted microbial genes.

Finally, a functional analysis is performed, focusing on the genes affected by each miRNA within each microbial genome. HolomiRA utilizes the SUPER-FOCUS software (v. 1.4.1) [27] for functional annotation, employing DIAMOND (v. 2.1.8) [28] as the aligner to achieve this.

Step 6 – Comparative analysis.

The pipeline automatically performs comparative analyses across different sample categories, such as phenotypes, case–control groups, or environments within the same host species, if more than one sample category is specified in the config.yaml file. The same summary metrics and functional enrichment outputs described in Steps 4 and 5 are generated, with results organized by category group, thereby allowing better visualization of the observed differences. To illustrate the overlap between groups, HolomiRA automatically generates Venn diagrams and a complementary text file listing unique and shared elements across categories, covering microbial taxa (at the species level), genes, and miRNAs. Histogram plots summarizing the unique counts of miRNAs, microbiome genes, microbiome genomes, and taxonomic groups within each category are also produced.

Additional Step 1 – Functional enrichment analysis: additional plot visualization and differential abundance testing.

Supplementary scripts provided with the pipeline allow users to generate plots highlighting the most abundant functions. For studies involving different sample groups (or datasets, *e.g.,* different species or experimental conditions), the pipeline includes a comparative statistical analysis focused on functional abundances. By default, functions are filtered based on two criteria: a minimum relative abundance threshold of 0.01 and a prevalence threshold of 20% (*i.e.,* functions must be present in at least 20% of the samples to be considered for analysis). After filtering, functions shared between the groups being compared are selected for differential abundance testing. The statistical analysis is performed using the Wilcoxon rank-sum test. Additionally, log_2_ fold change (log_2_FC) values are calculated to assess the magnitude of the differences. By default, functions are considered significantly differentially abundant if they meet both of the following criteria: an adjusted *p*-value ≤ 0.05 and |log_2_FC|> 1. *P*-values are adjusted for multiple testing using the Benjamini–Hochberg method [29].

Additional Step 2 - Comparative analysis between different results sets.

HolomiRA also supports comparative analyses between two independent result sets, generating Venn diagrams and histograms. The analysis identifies unique and shared elements across groups, including microbial taxa, genes, and miRNAs, and summarizes these findings in both graphical and tabular formats. The Venn diagrams illustrate overlaps, while histograms provide quantitative summaries of the unique counts of miRNAs, microbial genes, genomes, and taxa within each group.

## Real data

We evaluated HolomiRA using two publicly available datasets of metagenome-assembled genomes (MAGs) derived from human feces [30] and bovine feces and ruminal content [31]. The human dataset included 39,913 high-quality MAGs, and the ruminant dataset contained 10,000 high-quality MAGs, as defined by their respective original publications. No additional quality filtering was applied in our analysis. To reduce redundancy and optimize computational performance, we filtered the datasets to retain a single representative MAG per species or genus (for those classified only up to the genus level).

After filtering, we analyzed a total of 184 MAGs from human feces, and 118 and 140 MAGs from bovine feces and ruminal content, respectively. The list of MAGs used, as well as their taxonomic classification, can be found in Additional Table 1. We downloaded human and bovine miRNA sequences from the MirGeneDB database [23], which contains manually curated and experimentally validated miRNA genes. In total, 630 human miRNAs and 481 bovine miRNA sequences were included in our analyses (Additional file 1 and Additional file 2).

We performed the analysis using the HolomiRA pipeline with default parameters, except for the differential abundance testing, where we used a prevalence threshold > 0.05 and an adjusted *p*-value < 0.1. Average run times and the maximum memory used for each pipeline rule, obtained using the example files (available with the pipeline), can be found in Supplementary Table 2.

## Results and discussion

### Comparing bovine feces and rumen environment

**Fig. 2:**
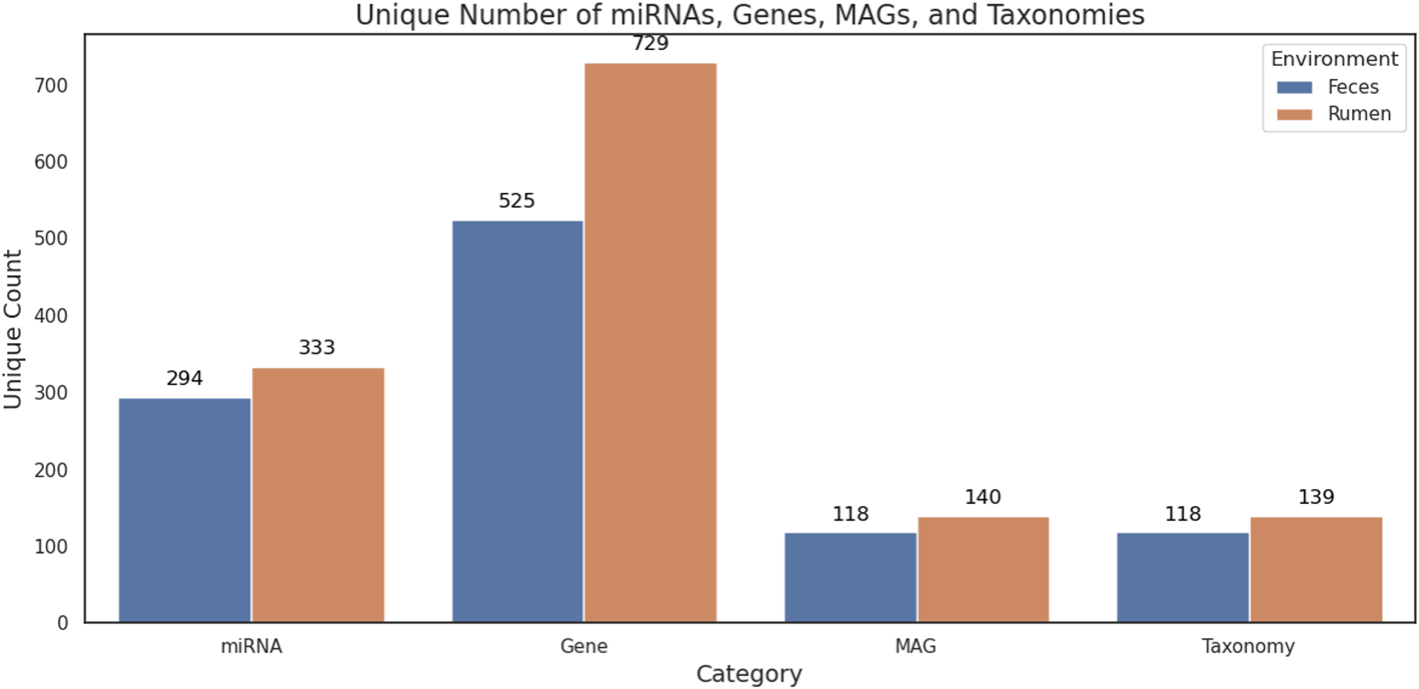
Unique counts of host miRNAs, putative microbial target genes, metagenome-assembled genomes (MAGs), and taxonomic units identified in each environment (bovine rumen and feces) according to HolomiRA. The *taxonomy* category refers to the number of unique microbial taxa detected at the species level

**Fig. 3:**
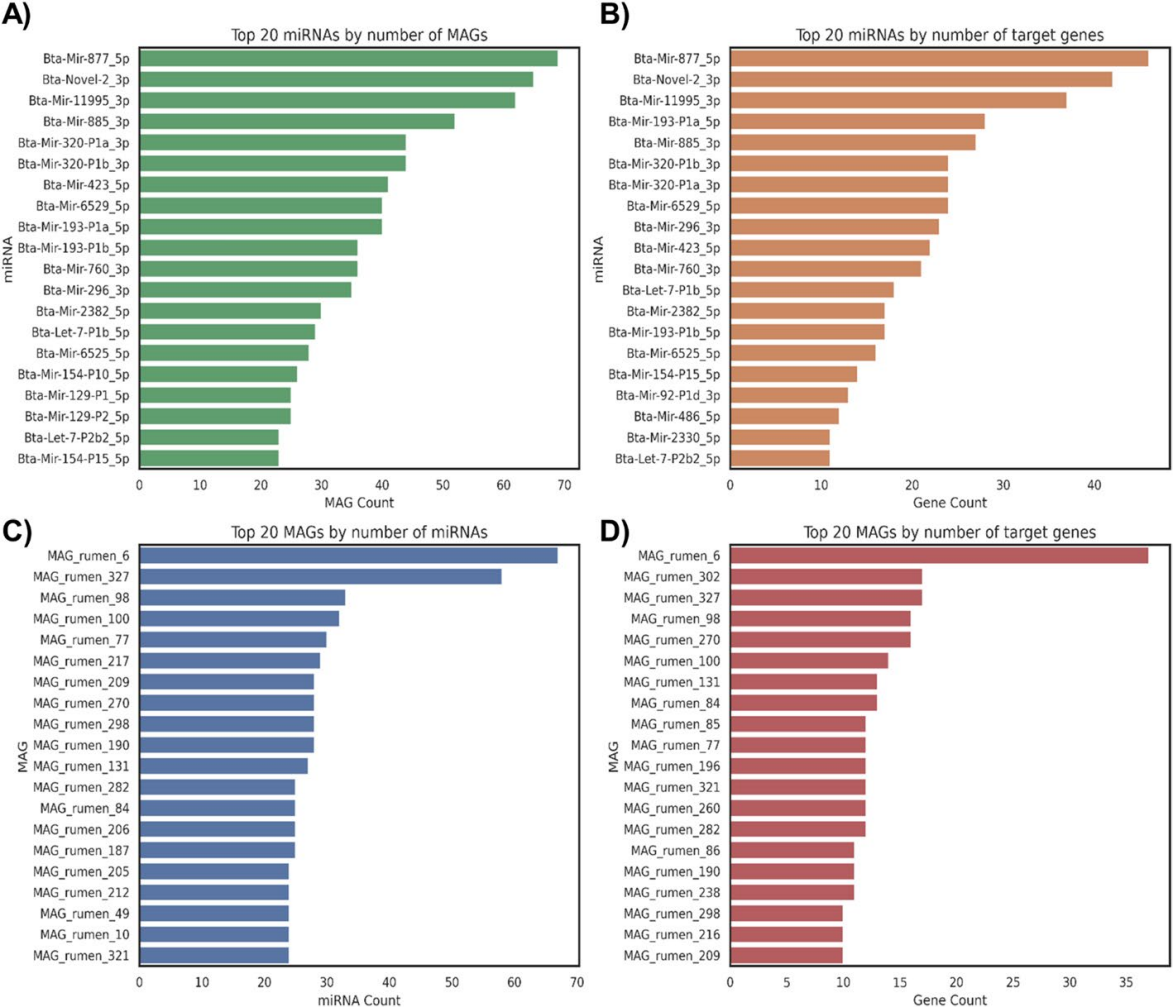
Top 20 features identified in the bovine ruminal content based on the number of predicted interactions. **A** Top 20 miRNAs ranked by the number of MAGs with predicted targets; **B** Top 20 miRNAs ranked by the number of affected target genes; **C** Top 20 MAGs ranked by the number of interacting miRNAs; and **D** Top 20 MAGs ranked by the number of predicted target genes

**Fig. 4:**
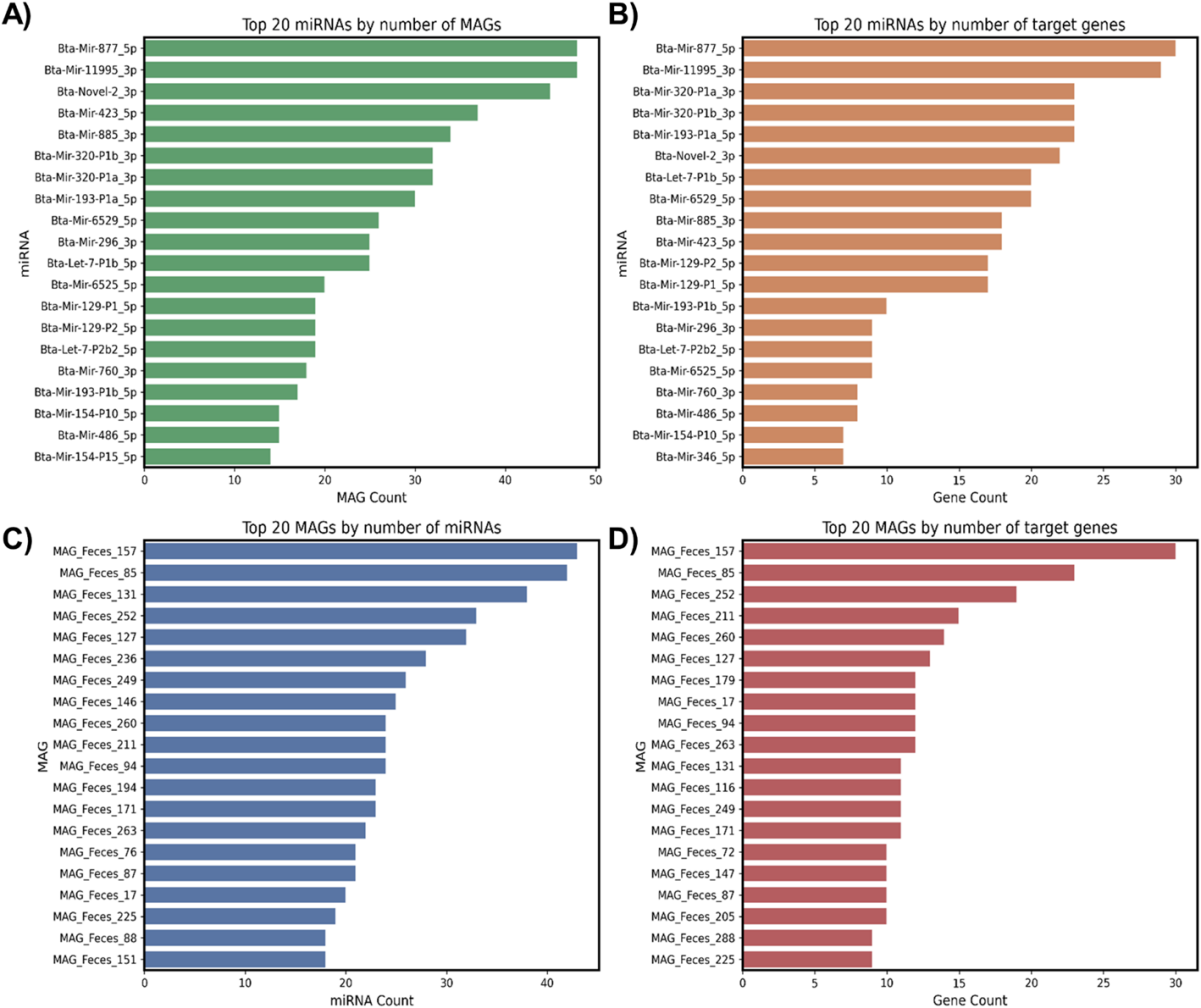
Top 20 features identified in the bovine feces based on the number of predicted interactions. **A** Top 20 miRNAs ranked by the number of MAGs with predicted targets; **B** Top 20 miRNAs ranked by the number of affected target genes; **C** Top 20 MAGs ranked by the number of interacting miRNAs; and **D** Top 20 MAGs ranked by the number of predicted target genes.

In our real dataset, we identified 333 and 294 miRNAs as putative candidates for binding to 729 and 525 bacterial genes in bovine ruminal content and feces, respectively (Fig. 2). In addition, HolomiRA enables the identification of miRNAs that affect the largest number of genomes (Figs. 3 and 4), as well as the specific genes they target. In the ruminal environment (Fig. 3A), Bta-Mir-877_5p (N = 69 MAGs), Bta-Novel-2_3p (N = 65 MAGs), and Bta-Mir-11995_3p (N = 62 MAGs) showed a broad distribution across MAGs, and also stood out for the number of predicted target genes. Bta-Mir-877_5p was associated with 46 putative target genes, Bta-Novel-2_3p with 42, and Bta-Mir-11995_3p with 37, suggesting a potential central regulatory role (Fig. 3B).

In the fecal environment (Fig. 4A), the same miRNAs (Bta-Mir-877_5p, Bta-Mir-11995_3p, and Bta-Novel-2_3p) were also among the most widely distributed, each targeting more than 40 MAGs. Additionally, Bta-Mir-877_5p (N = 30) and Bta-Mir-11995_3p (N = 29), along with Bta-Mir-320-P1a-3p (N = 23), Bta-Mir-320-P1b-3p (N = 23), and Bta-Mir-193-P1a-5p (N = 23), showed the highest number of putative target genes (Fig. 4B), which may indicate a conserved functional role across different compartments of the digestive tract.

Bta-Mir-877 has been implicated in the development of *Staphylococcus aureus-*induced mastitis [32] and Bta-miR-193a has been described as having antiviral activity [33]. Nana et al. [33] reported that this miRNA promotes apoptosis and inhibits the replication of bovine viral diarrhea virus. The bta-miR-320a is involved in the regulation of immunological pathways, such as AMP-activated protein kinase (AMPK) and Tumor Necrosis Factor (TNF), supporting a role in the host immune response [34]. In addition to these antiviral and immunoregulatory functions, miR-877, miR-193a, and miR-320a have been identified in milk exosomes, which are capable of surviving digestion and being absorbed by the intestinal tract, where they may interact with epithelial cells and microbiota [35]. Studies demonstrate a direct role for exosomes in microbial control [3, 36, 37]. Zhou et al. [37] observed that bovine milk content in the diet can alter the abundance of specific bacterial populations within the gut in C57BL/6 mice [37].

**Fig. 5:**
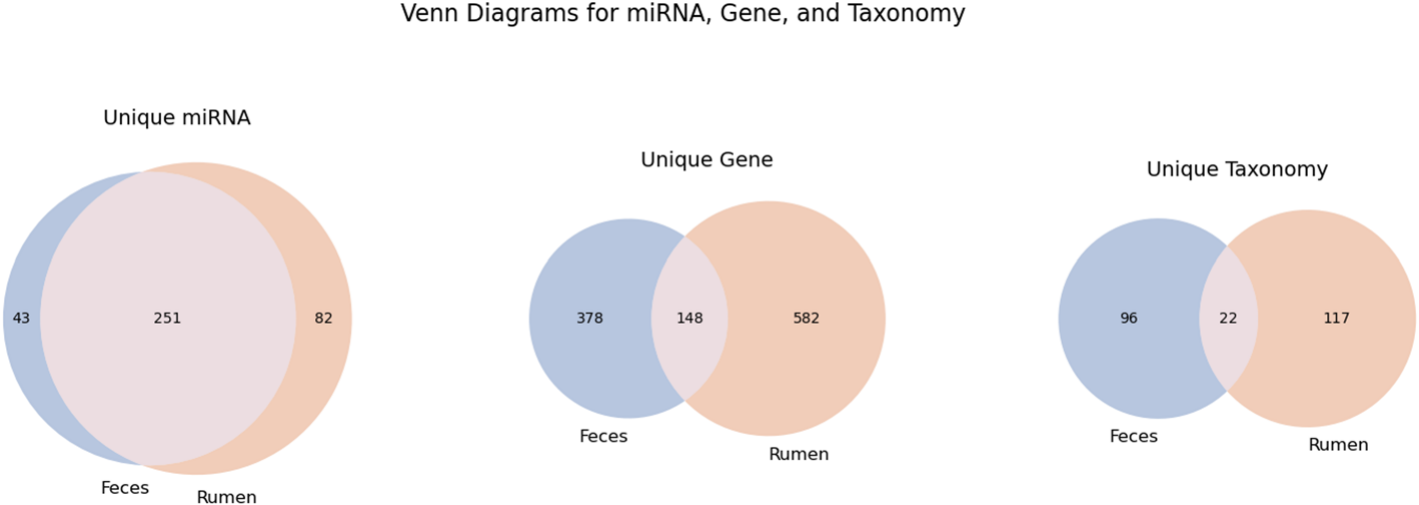
Venn diagrams illustrating shared and unique miRNAs, putative target genes, and taxonomic groups across bovine feces and ruminal content. Overlapping regions represent common elements, while non-overlapping areas highlight specific features that are unique to each environment

The pipeline also identifies lists of miRNAs and target genes that are specific to individual groups (Fig. 5). This analysis indicated that, despite the overlap of central miRNAs between the two environments, certain miRNAs and target genes may be exclusive to each microbiome. Our analysis identified 251 miRNAs shared between the two environments (Fig. 5). However, 43 miRNAs were exclusive to feces, while 82 were unique to the ruminal content (Fig. 5). This may suggest the presence of a common regulatory core, along with a subset of miRNAs specific to each niche, possibly reflecting distinct functional adaptations. Regarding putative target genes, the overlap was considerably smaller. Of the total, 148 genes were shared between both environments, while 378 were exclusive to the fecal microbiome and 582 were exclusive to the rumen (Fig. 5). This pattern indicates greater functional divergence between the microbiomes, as reflected in the set of putative genes regulated by miRNAs, and is consistent with the distinct digestive functions associated with each gut segment.

**Fig. 6:**
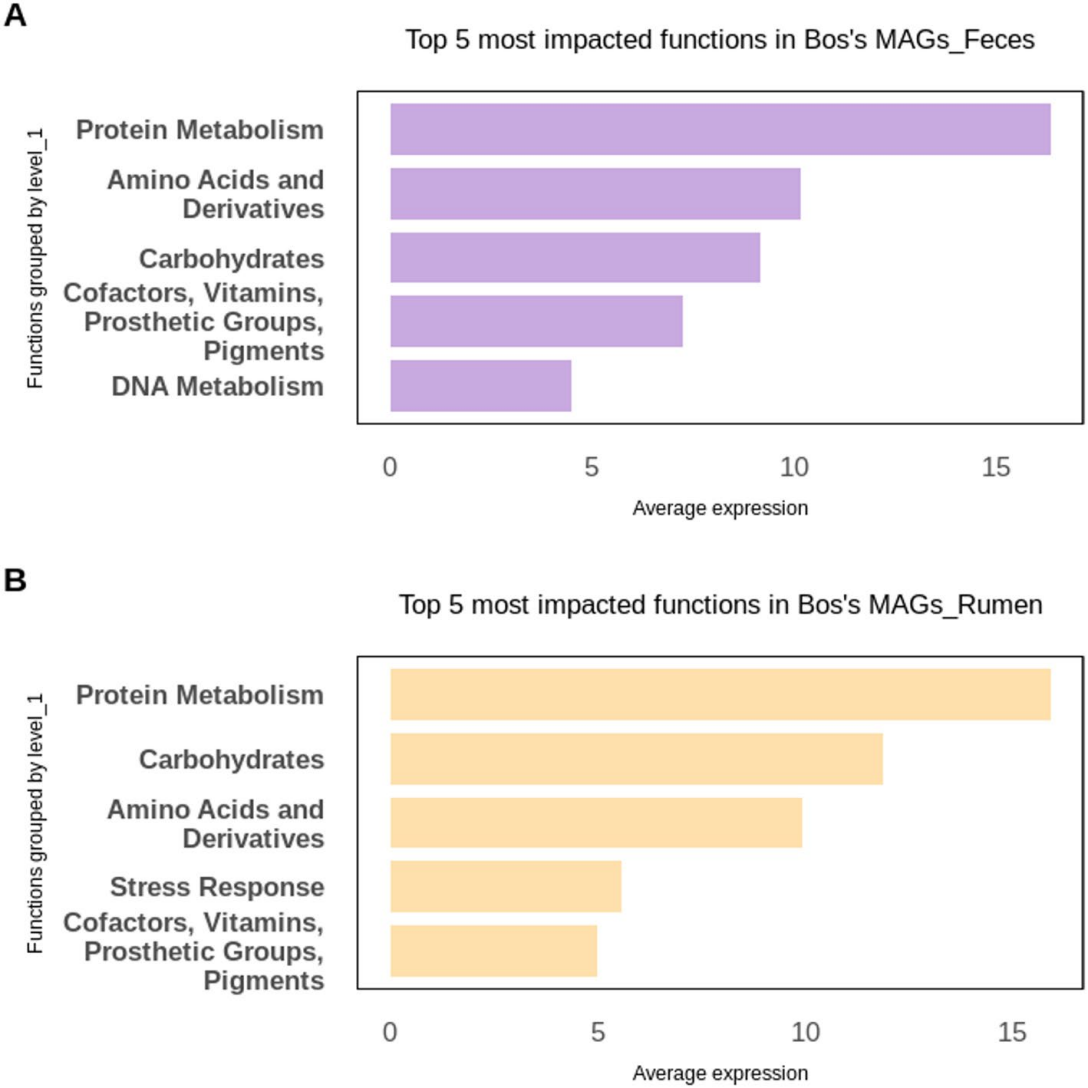
Most represented functional categories among the predicted microbial target genes in bovine microbiome. Functions are grouped at Level 1 of SUPER-FOCUS, based on the following biological datasets: **A** Bovine MAGs from feces, **B** Bovine MAGs from ruminal content

Functional analysis of genes putatively impacted by host miRNAs revealed that, in both bovine rumen and feces, functions related to protein metabolism, amino acids and derivatives, and carbohydrates were consistently represented (Fig. 6 and B). In the feces (Fig. 6A), functions linked to DNA metabolism were also enriched, suggesting alterations in recycling processes and final degradation activities [38]. In the rumen (Fig. 6B), stress response functions were enriched, indicating potential effects on microbial fermentative roles and their adaptation to variable environmental conditions [39, 40].

**Fig. 7:**
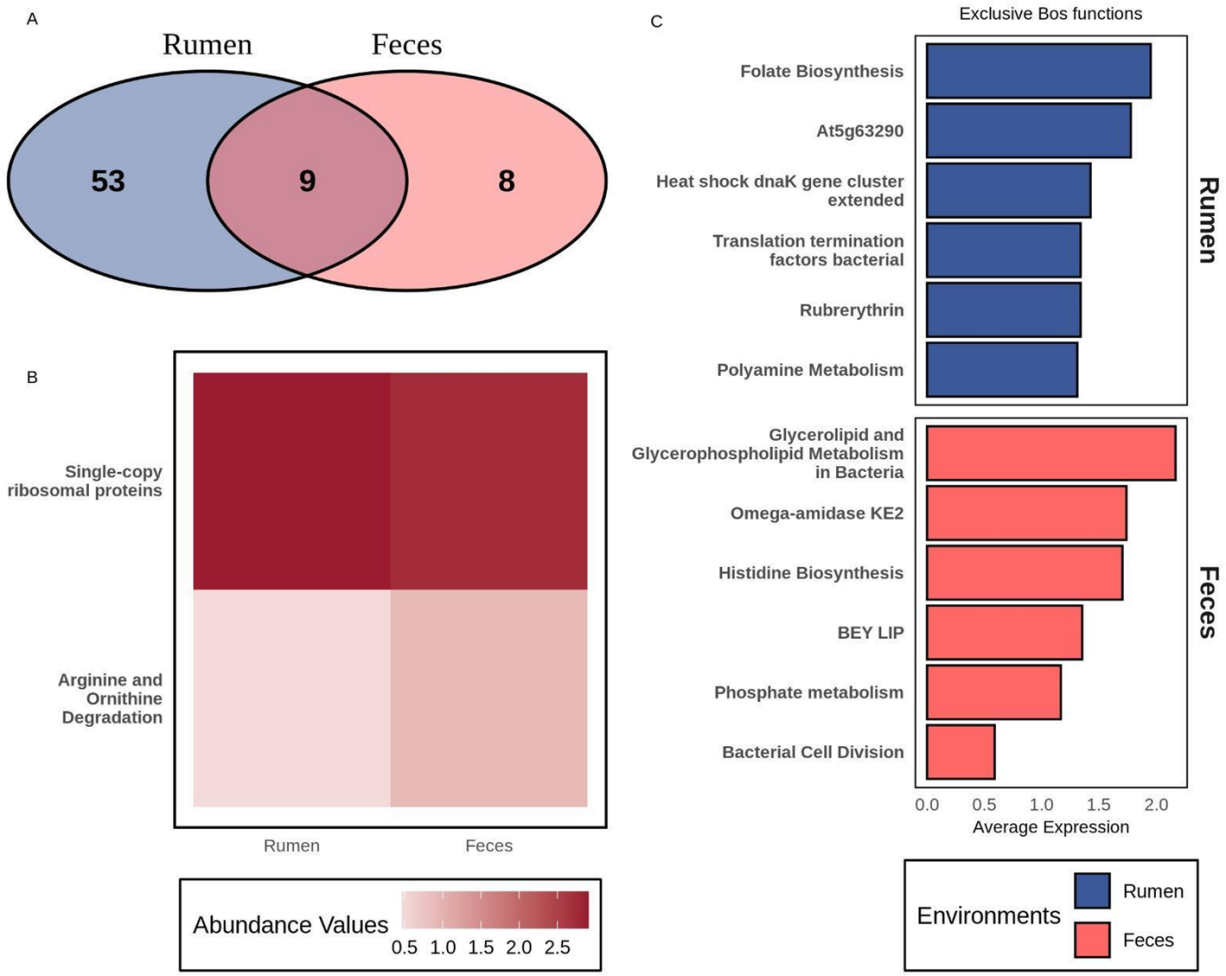
Functions grouped at SUPER-FOCUS level 3 among putative host miRNA-impacted genes from metagenome-assembled genomes (MAGs) obtained from bovine feces and rumen. Red and blue bars represent functions from bovine feces and rumen, respectively. **A** Venn diagram showing functions predicted to be unique to each environment and those shared between the environments. **B** Heatmap plot showing differentially abundant functions between bovine feces and rumen. **C** Barplot highlighting functions predicted to be exclusive to each environment

In Fig. 7, we present a comparative analysis of functions, grouped at SUPER-FOCUS level 3, related to MAG genes predicted to be affected by host miRNAs in the bovine feces and rumen. The Venn diagram shown in Fig. 7A revealed nine functions shared between the two biological datasets. Regarding unique functions, eight were exclusive to the bovine fecal microbiome, while 53 were unique to the ruminal environment (Fig. 7A). This difference suggests that the rumen microbiome is functionally more diverse, which is expected given its central role in anaerobic digestion of complex polysaccharides and the fermentation of fibrous substrates [41, 42].

Furthermore, statistical analysis revealed that two of the nine shared functions were differentially abundant, as illustrated in Fig. 7B. Functions related to single-copy ribosomal proteins were more impacted in the rumen than in bovine feces, indicating a higher rate of microbial growth and metabolic activity in the ruminal environment. This finding is consistent with the rumen’s requirement to support a highly active microbial community for rapid feed degradation [43, 44]. In contrast, the lower abundance observed in feces could reflect a microbiota more adapted to limited substrate availability and intestinal transit. Figure 7C provides a detailed view of the top eight most abundant functions identified in bovine feces and rumen. These exclusively impacted functions further support the concept of environmental specialization. In the rumen, higher representation of functions related to folate biosynthesis, polyamine metabolism, and response to heat stress was observed, indicating possible adaptations to maximize bacterial growth and to protect against stress conditions, such as pH and temperature variations during fermentation [45, 46]. In feces, functions related to glycerolipid metabolism and histidine biosynthesis were more prominent, suggesting adaptation to an environment with lower nutrient availability and increased microbial competition [47, 48].

### Comparing bovine and human feces

**Fig. 8:**
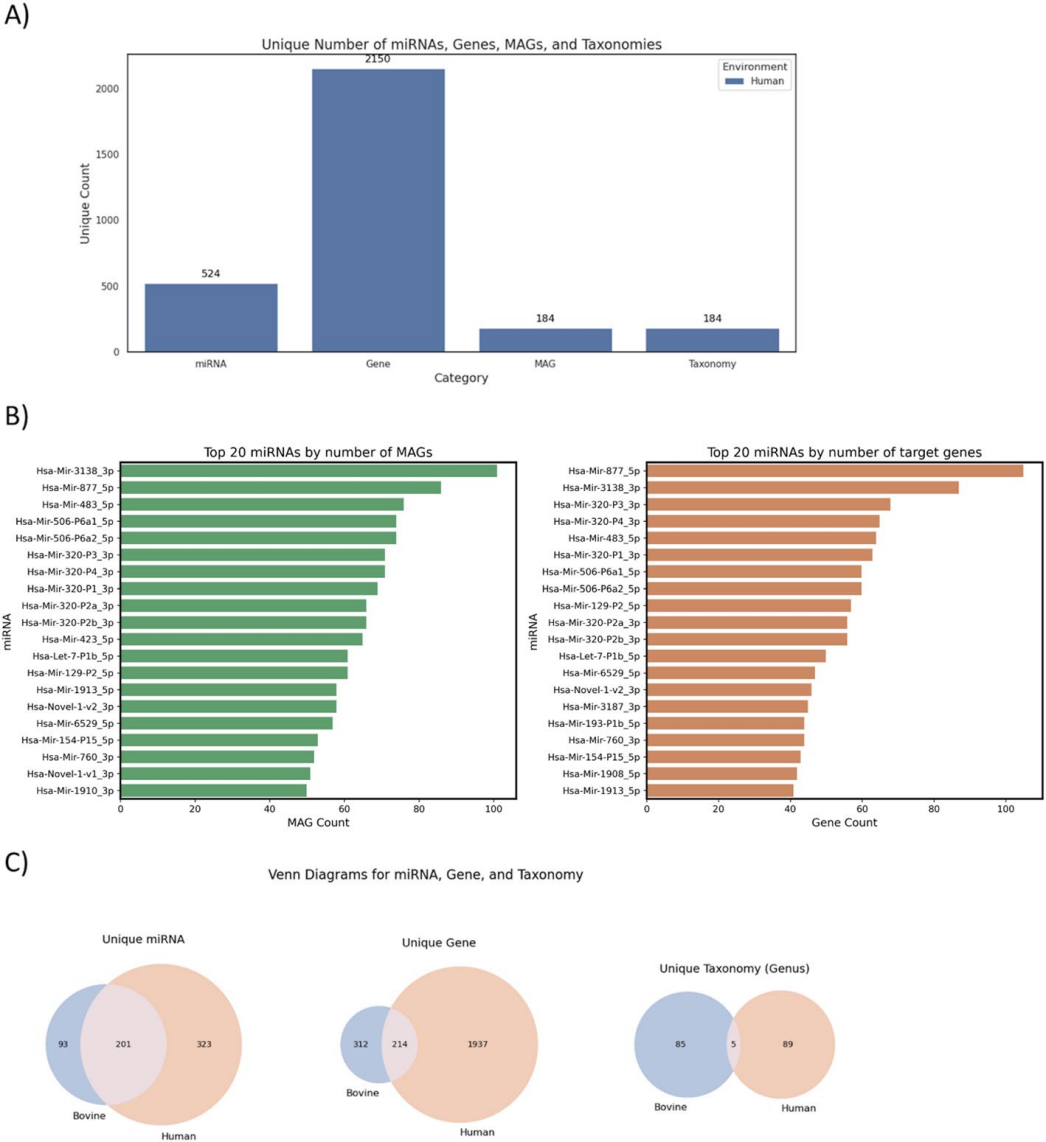
Predicted host miRNA-microbiome interactions in human fecal metagenome-assembled genomes (MAGs) according to HolomiRA. **A** Unique counts of host miRNAs predicted to target the microbiome, putative microbial target genes, miRNA-targeted MAGs, and taxonomic units identified in human fecal MAGs. The *taxonomy* category refers to the number of unique microbial taxa detected at the species level. **B** Top 20 miRNAs ranked by the number of associated MAGs (left) and the number of predicted miRNA target genes (right) in MAGs from human feces. **C** Venn diagrams comparing the overlap of unique miRNAs, genes, and taxonomic genera between MAGs from bovine and human feces

In total, 524 miRNAs were predicted to impact 2,150 genes in human fecal MAGs (Fig. 8A). Hsa-Mir-3138_3p (N = 101), Hsa-Mir-877_5p (N = 86), and Hsa-Mir-483_5p (N = 56) were predicted to impact the highest number of MAGs. At the same time, Hsa-Mir-877_5p (N = 105), Hsa-Mir-3138_3p (N = 87), and Hsa-Mir-320-P3_3p (N = 68) stood out for having the highest number of putative target genes, suggesting their potential central role in microbiota gene regulation in the human gut (Fig. 8B). Recently, Gao et al., [49] reported, using a dual-luciferase reporter assay, that miR-320 can directly target the *ORF6* gene of porcine reproductive and respiratory syndrome virus (PRRSV), inhibiting PRRSV replication. In addition, miR-320a was identified as a potential blood-based biomarker for tuberculosis, with potential utility for diagnosing drug-resistant forms of the disease [50].

In addition, Venn diagrams were used to compare the overlap of miRNAs, genes, and taxonomic groups between the putative miRNA impact results from human and bovine MAG genes (Fig. 8C). A total of 201 miRNAs, 214 genes, and 5 taxonomic genera were shared between the two environments. However, the presence of unique elements was also evident, indicating host-specific diversity. In particular, human MAGs showed a substantially higher number of unique genes (n = 1937) compared to bovine samples (n = 312), which may reflect differences in functional complexity or microbial gene expression profiles.

**Fig. 9:**
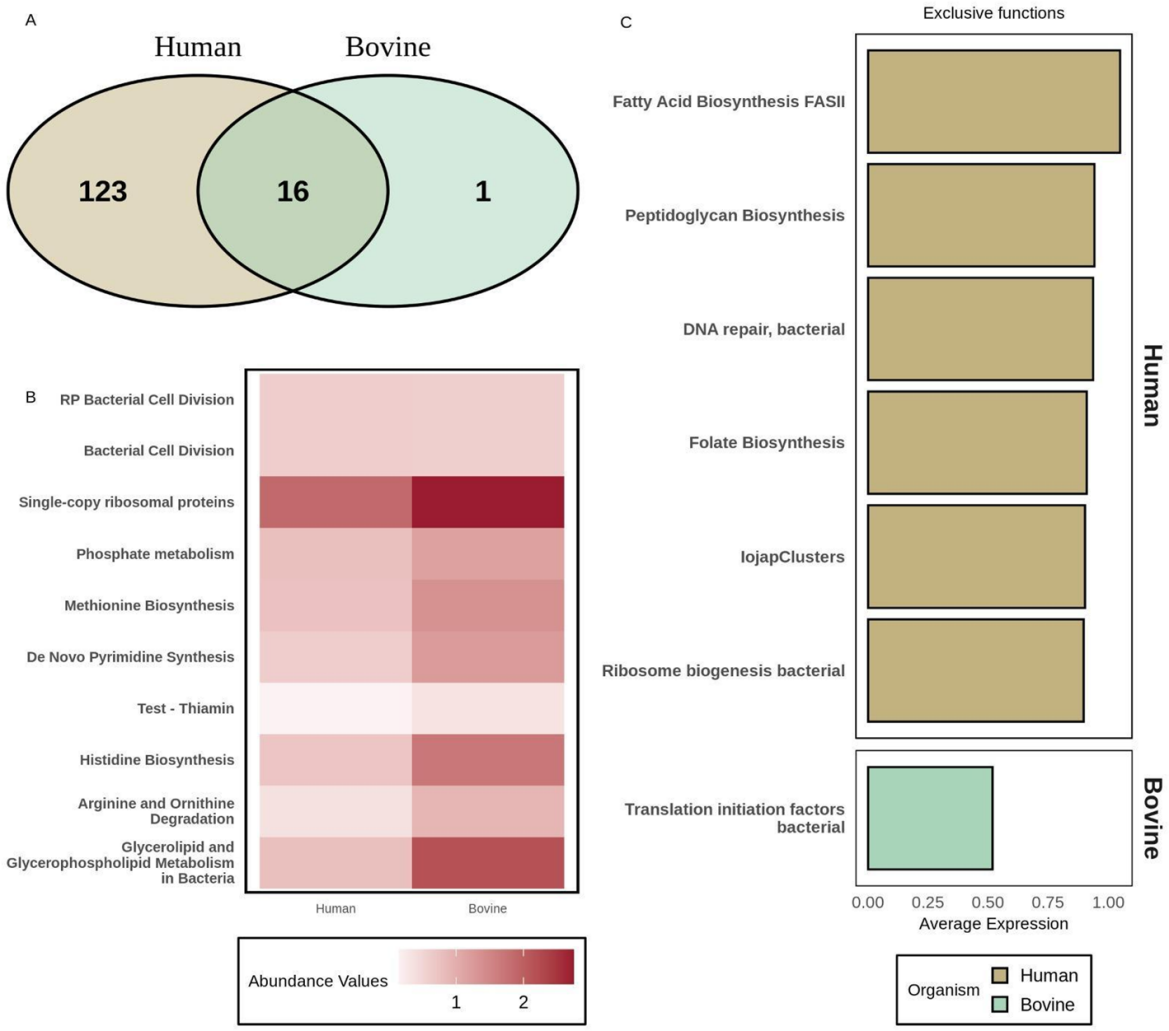
Functions grouped at SUPER-FOCUS level 3 among putative host miRNA-impacted genes from metagenome-assembled genomes (MAGs) obtained from bovine and human feces. Brown and green bars represent functions from human and bovine feces, respectively. **A** Venn diagram showing functions predicted to be unique to each environment and those shared between the two host species. **B** Heatmap plot showing differentially abundant functions between bovine and human feces. **C** Barplot highlighting functions predicted to be exclusive to each host species

Figure 9 presents a comparative functional analysis of putative miRNA-impacted genes in fecal MAGs from human (in brown) and bovine (in green) samples, grouped at SUPER-FOCUS level 3. The Venn diagram in Fig. 9A revealed 16 functions shared between the two host species, out of a total of 140 identified functions. Furthermore, statistical analysis indicated that 10 functions were differentially abundant between the two groups, as illustrated in Fig. 9B. In this figure, a heatmap illustrates the relative abundance of differentially abundant functions between the two host species. Compared to human fecal MAG genes, the higher impact on functions such as bacterial cell division, methionine and histidine biosynthesis, and arginine/ornithine degradation observed in bovine fecal MAG genes may be associated with dietary and physiological differences. In bovines, the fibrous diet and ruminant digestive structure favor the accumulation of nitrogenous residues in the hindgut, requiring increased microbial activity for biomass synthesis and competition for resources, which is reflected in the importance of these functional pathways [51, 52].

Regarding unique functions, bovine feces exhibited only one, while human feces contained 123 unique functions. Figure 9C shows this single bovine-specific function along with the top six functions predicted to be exclusive to human feces. The greater predicted impact of miRNAs on genes retrieved from human feces MAGs, as well as the presence of functional pathways unique to the human fecal microbiome, can be attributed to differences in digestion and diet between the two host species. Unlike bovines, humans possess a less fermentative gut and typically consume a diet rich in preformed nutrients, such as amino acids and vitamins. This may reduce the selective pressure for microbial biosynthesis and increase the sensitivity of these pathways to post-transcriptional regulation. This environmental context can favor bacterial taxa with high demands for growth and repair pathways, including folate and peptidoglycan biosynthesis, as well as ribosome biogenesis. These functions might represent sensitive targets for regulation by fecal miRNAs, which have already been shown to modulate essential bacterial genes [4, 53].

## Conclusion

HolomiRA is a user-friendly and reproducible pipeline for predicting host-miRNA target genes in prokaryotic genomes, with the aim of exploring potential host-microbiome regulatory interactions. By integrating gene annotation, target-site prediction, and functional analysis, the pipeline provides a framework for identifying the putative roles of miRNAs in shaping microbiome dynamics and function. HolomiRA employs a hybridization-based prediction strategy and restricts target search windows to regions surrounding the RBS. This methodical approach prioritizes biologically plausible and energetically stable interactions. However, it is important to note that the primary objective of HolomiRA is to facilitate hypothesis generation. Therefore, in downstream analyses, we strongly recommend the implementation of complementary validation strategies, such as the use of independent datasets or experimental assays, to confirm predicted interactions and strengthen biological interpretations.

## Availability and requirements

Project name: HolomiRA.

Project home page: https://github.com/JBruscadin/HolomiRA.

Operating system(s): This workflow has been tested on linux systems.

Programming language: Snakemake pipeline, Python, Bash and R.

Other requirements: Dependencies installed via Conda/Mamba.

License: GNU GPL.

Any restrictions to use by non-academics: None.

## Supplementary Information

Below is the link to the electronic supplementary material.

Supplementary Material 1: File 1 List of cattle miRNAs tested by HolomiRA

Supplementary Material 2: File 2 List of human miRNAs tested by HolomiRA

Supplementary Material 3: Table S1 Metagenome-assembled genomes (MAGs) derived from human feces, bovine feces, and bovine ruminal content analyzed using HolomiRA. Table S2 Running time and memory consumption of the HolomiRA workflow using test data.

## Data Availability

Pipeline code and manual are available at https://github.com/JBruscadin/HolomiRA. Data for benchmarking were drawn from cited papers. The complete human feces MAG dataset is available at ftp://ftp.ebi.ac.uk/pub/databases/metagenomics/hgg_mags.tar.gz, and the complete bovine gastrointestinal MAG dataset is accessible through the European Nucleotide Archive (ENA) database, project PRJNA657473.
